# The Influence of Ethynyl In-Chain Crosslinkers on the Properties of 6FDA-Based Polyimides

**DOI:** 10.3390/ma16010169

**Published:** 2022-12-24

**Authors:** Valeri Ivanov Petkov, Leonardo Pelcastre, Carlos Solano, Patrik Fernberg

**Affiliations:** 1 Department of Engineering Sciences and Mathematics, Luleå Tekniska Universitet, 971 87 Luleå, Sweden; 2 Nexam Chemical AB, 234 35 Lomma, Sweden

**Keywords:** polyimide, thermal oxidative degradation, fracture toughness, DSC, nanoindentation

## Abstract

Two 4,4′-(hexafluoroisopropylidene)diphthalic anhydride-based thermosetting polyimide formulations with varied amounts of crosslinking sites were compared to understand the influence of crosslinking density on fracture toughness, glass transition temperature and thermal oxidative stability. The thermal and mechanical properties of both materials were investigated through a series of single-edge notched beams, differential scanning calorimetry, dilatometry, weight loss, light optical microscopy and nanoindentation experiments. It was found out that the reduced crosslinking resulted in slightly increased fracture toughness but decreased the *T*g of the material. No significant difference could be observed in the thermal oxidative stability with the experimental techniques considered.

## 1. Introduction

NEXIMID MHT-R is a high-end thermosetting polyimide that is particularly suitable for composite manufacturing through resin transfer moulding (RTM), due to its low melt viscosity prior to crosslinking. Previous studies have shown that MHT-R can reach a glass transition temperature (*T*g) between 340–466 °C, making it an interesting light-weight material for high-temperature applications [1,2]. The presence of oxygen, in addition to high temperature, however, reduces the ability of the polymer to withstand damage, resulting in thermal oxidative degradation, or thermal oxidation. The complex interaction between temperature, oxygen diffusion, absorption, and reaction kinetics during thermal oxidative degradation of thermally stable polymers, such as polyimides, has been described by Schoeppner et al. [3].

Previous studies have also shown that additional factors that affect the thermal oxidative behavior of carbon fiber/polyimide composites are the susceptibility of the polymer towards chemically reacting with oxygen-rich air at elevated temperatures, and matrix cracking [3,4]. The first factor usually results in an oxidation layer with reduced mechanical properties on the exposed surfaces, while the second one creates direct access for oxygen into the specimen, thus shortening the amount of distance the oxygen must diffuse through. Cracking can occur due to the presence of residual stresses from manufacturing but can also be exacerbated by the locally reduced properties in the oxidation layer.

This study introduces a new polyimide resin formulation, NEXIMID R300, with potential to reduce both matrix cracking and thermal oxidation, while retaining RTM processability, by introducing a change in the chemical composition of the original MHT-R matrix. The idea behind the modification of the polyimide resin is to lower the crosslink density by reducing the internal crosslinking units, thus enhancing the toughness of the material and the resistance to cracking when used in composites. Another consequence of the reduced crosslink density could be an improved thermal oxidative stability since the polyimide backbone is considered more stable under conditions that promote thermal oxidation than the structure created by the polymerization of the oligomers.

The aim of this work is to investigate and compare the novel NEXIMID R300 to the original MHT-R in terms of thermal and mechanical properties, such as *T*g, thermal expansion, ageing behavior, fracture toughness, Young’s modulus and hardness, for which a series of differential scanning calorimetry (DSC), dilatometry, weight loss, light optical microscopy (LOM), single-edge notched beam (SENB) and nanoindentation experiments were performed. Measuring the fracture toughness on bulk specimens of high-crosslinking-density polyimides that are also RTM-processable is a challenging experiment due to the inherent brittleness of these materials, and to the knowledge of the authors this is one of the first studies of this kind.

## 2. Materials and Methods

**Figure A1:**
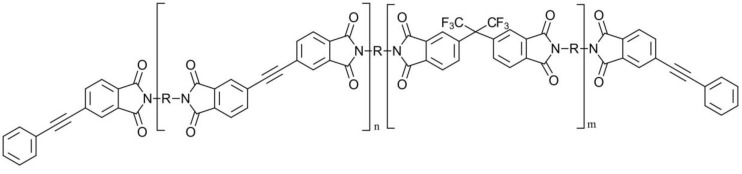
A generic representation of the polyimide structure, including EBPA as an in-chain crosslinker.

*Materials:* Two 4,4′-(hexafluoroisopropylidene)diphthalic anhydride-based thermosetting polyimide formulations (6FDA PI) were considered in the study. The first PI formulation contained a 6FDA backbone, with a 4-(phenylethynyl)phthalic anhydride (4-PEPA) end-group crosslinker and an ethynyl bis-phthalic anhydride (EBPA) main chain crosslinker. A schematic figure on the generic structure, including EBPA crosslinker, can be found in Appendix A, Figure A1. Studies on fiber-reinforced composites based on this resin formulation are available [1,2] and the resin is made available by Nexam Chemicals AB, Lomma, Sweden, under the tradename NEXIMID^®^ MHT-R. In the second formulation, NEXIMID^®^ R300, henceforth labelled R300, all fully aromatic monomers were preserved compared to MHT-R, except for the in-chain crosslinker EBPA, which accounted for around 5% of the ethynyl crosslinking units and was removed to reduce the crosslinking density upon curing. The concentration of ethyl containing monomers was decreased from 42 mol% in MHT-R to 39 mol% in R300. It should also be considered that compared to cross-linkers attached to the end of the polymer chain, i.e., reactive end-cappers, crosslinkers incorporated in the inner part of the polymer chain, i.e., in-chain crosslinkers, are more effective in promoting crosslinking since they do not have the possibility to engage in linear chain extension events.

*Plate manufacturing and specimen preparation:* The two PI resin systems were used for manufacturing two separate plates with dimensions 350 × 350 × 3 mm^3^. The plates were manufactured by RTM at RISE Sicomp, Öjebyn, Sweden. The manufacturing parameters were like the previously used settings in [1], including a two-step curing cycle at 320 °C (for 30–60 min) and 370 °C (for 120–180 min). Manufacturing of the 3 mm-thick neat PI plates is not a trivial process, due to the stresses generated under the high temperature curing cycle and the lack of reinforcement. To tackle these issues, the plates were designed to include a carbon fiber preform based on satin weaves as a frame around a centrally located cavity, which was left without fibers. The square cavity section contained a coarse steel wire network that served the purpose of controlling crack initiation and propagation. The neat polymer section with MHT-R was 160 × 160 mm^2^ and was increased to 240 × 240 mm^2^ for the plate made with the new R300 polyimide formulation. For the carbon fiber-reinforced frame part of the plates, 370 g/m^2^ 8-harness satin weave based on Cytec Thornel T650/35 carbon fibers (Sigmatex Ltd., Runcorn, UK) were used.

Neat (non-reinforced) polymer specimens were cut out from the RTM-manufactured plate with a Dremel cutting device, equipped with a diamond cut-off wheel, and were grinded with sandpaper to the required dimensions for each respective test.

**Figure 1:**
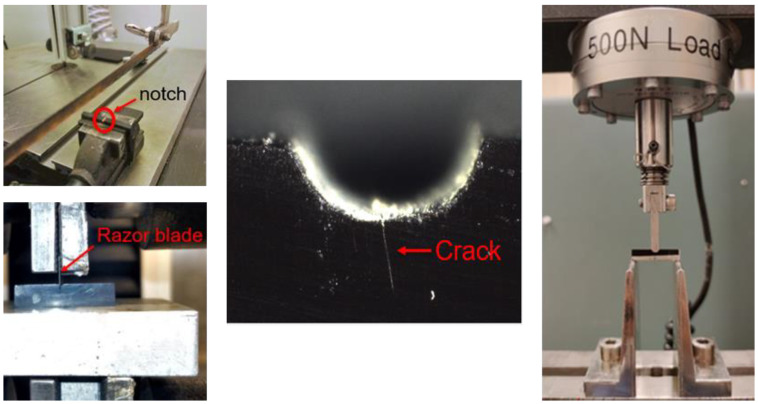
Fracture toughness sample preparation and experiment setup.

*Fracture toughness:* Fracture toughness measurements were performed on SENB specimens according to ASTM D5045-14 [5]. Specimen preparation involved creating a shallow notch with a fine saw, while a razor blade mounted in an Instron 3366 tensile testing machine was used to create the sharp initial pre-crack by controlled penetration into the specimen. The specimen dimensions were 26 × 5.8 × 3 mm^3^. Instron 4411 (Instron, Norwood, MA, USA) tensile testing machine with a 500 N load cell and three point bending setup were used for the experiments (Figure 1). The pre-crack initiation proved very challenging, especially for the MHT-R. Pre-cracks would often spontaneously propagate through the whole specimen, as soon as it had been initiated by the razor blade, splitting the specimen in half. Due to the brittleness of the material, the notch length recommended in the standard could not be achieved for samples based on the original MHT-R formulation. To ensure that the initial crack was extending through the whole width of the specimen, a Nikon SMZ1270 (Nikon, Tokyo, Japan) stereomicroscope was used to investigate the pre-cracks, as shown in Figure 1, before and after the test. Another criterion that specimens had to fulfil to be considered adequate was that their load-extension curves from the SENB tests had to be free from any artefacts, which would suggest, e.g., faulty pre-crack geometry or porosity on the path of the crack. Eight correctly pre-cracked R300 specimens were successfully prepared. Four of them resulted in a successful test. For MHT-R, three specimens met the set of conditions.

*DSC:* DSC experiments were performed in a Mettler Toledo DSC 821 to obtain thermal properties such as heat of reaction during curing and *T*g for both polymer formulations. The uncured polymer powder was dried in an oven for one hour at 90 °C before the DSC experiments. The DSC procedure consisted of three heating steps with cooling down to 25 °C between each step: 25–200 °C at 10°C/min, 25–500 °C at 10 °C/min, 25–500 °C at 20 °C/min.

*Dilatometry and Coefficient of thermal expansion:* Netzsch DIL 402 C dilatometer was used for obtaining data for calculating the coefficient of thermal expansion (CTE) of both polymers in their cured state, according to ASTM E831-19 [6]. The experiments were performed under a nitrogen environment and consisted of a temperature ramp from 25 °C to 450 °C with a heating rate of 5 °C/min.

*Ageing, weight measurements, LOM:* The samples from each plate were aged isothermally in a Nabertherm N11/HR (Nabertherm GmbH,. Lilienthal, Germany) furnace at a temperature of 288 °C for up to 1500 h (about 2 months) in ambient air. The dimensions of the samples were approximately 12 × 6 × 3 mm^3^. The weight was measured at time intervals of 0, 4, 24, 72, 168, 336, 500, 1000 and 1500 h with a Mettler Toledo AG245 balance with an accuracy of 0.01 mg.

The weight loss, *W*, at time, *t*, was calculated according to Equation (1), where *m*_0_ is the initial mass before ageing and *m*(*t*) is the mass at a specific time.

(1) $$W\left(t\right)=\frac{m_{0}-m\left(t\right)}{m_{0}}\times 100\%$$

Additional specimens from both plates were prepared for LOM investigation. The dimensions were 12 × 6 × 3 mm^3^ and the specimens were aged for 72, 240, 500, 1000 and 1500 h under the same conditions as described previously. The specimens were mounted in epoxy and polished. The final polishing step was performed at 0.06 µm with Buehler MasterMet Colloidal Silica polishing suspension. A Nikon Eclipse MA200 light optical microscope supported with the Nikon NIS-Elements software were used for observing and measuring the oxidation layer thickness. To detect the oxidation layer, reflected light differential interference contrast (DIC) microscopy was used. The setup included a polarizer/analyzer unit (Nikon MA2-PA) and a Nomarski prism (Nikon L-DIC).

*Nanoindentation:* Room temperature nanoindentation tests were performed in a MicroMaterials NanoTest Vantage machine (Micro Materials Limited, Wrexham, UK), using a Berkovich tip. A maximum 50 mN load was used with a 2 s fixed loading and unloading time, with a dwell time of 600 s (10 min) at maximum load to account for the viscoelastic effect, following earlier studies on PMR-15 [7,8]. On each specimen, at least 15 indentations were made. On the aged specimens, at least 10 indentations were within the oxidation layer, approximately 50 µm from the edge, and five indentations were in the middle of the specimen, i.e., outside of the oxidation zone.

The reduced modulus (*E*_r_) was determined on the unloading segment of the hysteresis curve, starting at 100% and stopping at 40% of the maximum load. The Young’s modulus of the indented material (*E*_m_) was calculated directly in the NanoTest™ Vantage platform, using Young’s modulus (*E*_i_) of 1141 GPa and Poisson’s ratio (*υ*_i_) of 0.07 for the Berkovich indenter and Poisson’s ratio (*υ*_m_) of 0.38 [9] for the polyimide. The Young’s modulus of the indented material was obtained from the reduced modulus through Equation (2):

(2) $$\frac{1}{E_{r}}=\frac{1-\upsilon_{m}^{2}}{E_{m}}+\frac{1-\upsilon_{i}^{2}}{E_{i}}$$

During the indentation, the NanoTest continuously measures and records the penetration depth or displacement as a function of the applied load. Equation (3) shows the equation used for calculating the hardness (*H*) of the sample material:

(3) $$H=\frac{P_{\max}}{A_{C}}$$

where *P*_max_ is the peak load during indentation and *A_C_* is the cross-section area (also known as area function), which is used to calculate hardness according to the method of Oliver and Pharr [10].

A test schedule was performed on a fused silica sample to examine the state of the Berkovich tip. A load of 50 mN, a 2 s fixed loading and unloading time and a dwell time of 5 s were used. The obtained hardness and modulus values were 9.88 GPa (±0.163) and 75.7 GPa (±0.857), respectively, based on 7 indentations. The values were slightly higher than what has been previously obtained from the same sample (9 GPa and 71 GPa).

## 3. Results and Discussion

### 3.1. Fracture Toughness on Bulk Specimens

Table 1 compares the fracture toughness of isotropic neat resin specimens with a thickness of 3 mm. The average K_IC_ for R300 (0.88 MPa√m) was slightly higher than that for MHT-R (0.78 MPa√m) and could be correlated to the decrease in crosslink density, which was the main motivation behind the development of the new formulation. The material exhibits greater toughness than some epoxy resins, such as the RTM-optimized Hexcel RTM6 (0.62 MPa√m) and Araldite LY 564 (0.68 MPa√m) [11,12]. Compared to the toughened CYTEC PR520 epoxy resin for RTM, which is generally perceived as a very tough material (2.05 MPa√m) [13], the MHT-R and R300 are still relatively brittle. The studied polyimides have comparable K_IC_ values to other high *T*g thermosetting polymers [14]. The results gave an indication of enhanced fracture toughness but, due to the small sample size—three measurements on MHT-R and four on R300—the tests did not provide statistically secured evidence of actual difference and should not be considered conclusive. A carbon fiber composite plate with the R300 formulation as a base should be manufactured to further verify the improved fracture toughness.

### 3.2. DSC

DSC investigations were conducted to obtain information on the thermal properties such as heat of reaction and *T*g for the two resins. The DSC results confirmed the hypothesis that the new formulation should have lower crosslinking, reflected in the lower heat of reaction, and lower *T*g of the R300 polyimide (Table 2). There was a noticeable difference in the softening points and the uncured *T*g of the materials during the initial and the second heating ramps, respectively, which further underlined the differences in the basic form of the two formulations before curing.

The measured *T*g in the current study were around 405 °C for R300 and 432 °C for MHT-R. This is in the range, between 370 °C to 466 °C, that was reported earlier for MHT-R when determining *T*g for different curing conditions using DMTA [1]. Varying curing and post-curing cycles above 370 °C can be used to reach extremely high *T*g in MHT-R, but such manufacturing procedures might introduce damage into the composite and are not practically recommended. The curing of the DSC samples in the current study was conducted at temperatures of up to 500 °C during the non-isothermal program. Hence, it is reasonable to expect that the DSC-measured *T*g of the materials will be higher compared to a composite component, which was produced by the recommended RTM manufacturing schedule for carbon fiber/polyimide composites.

A difference of 27 °C in *T*g between the two formulations was observed, with MHT-R exhibiting the higher value. Crosslink density along with polymer back-bone structure rigidity, i.e., the tendency for segments involving multiple molecules to undergo coordinated conformation changes, are the two main aspects governing the *T*g of highly crosslinked thermosets. It was considered that the very small differences in actual molecular structure between the two grades were insufficient to cause large alterations to the polymer back-bone rigidity and hence this difference was attributed to lowered crosslinking densities between the two grades caused by the 5% reduction of crosslinking sites in R300.

### 3.3. Coefficient of Thermal Expansion

The CTE, Table 2, was calculated from the slope of the linear portion of the dilatometry curves, according to Equation (4):

(4) $$\alpha =\frac{1}{\Delta T}\frac{\Delta L}{L_{0}}$$

where Δ*T* is the temperature interval, Δ*L* and *L*_0_ are the elongation and the initial length of the specimen, respectively, giving the CTE, α, in µm m^−1^ °C^−1^. The average CTE values were 54.3 µm m^−1^ °C^−1^ for R300 and 56.0 µm m^−1^ °C^−1^ for MHT-R, indicating that there might be a small reduction in CTE in the R300. The values are comparable to the CTE of epoxies, which is around 55 µm m^−1^ °C^−1^.

### 3.4. Weight Loss and Oxidation Layer Measurements

**Figure 2:**
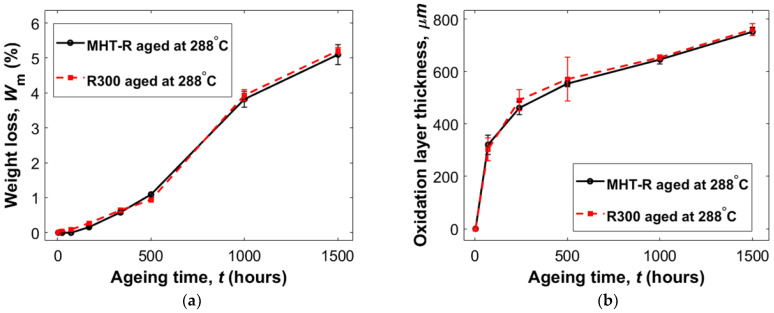
Weight loss (**a**) and oxidation layer thickness (**b**) measurements on MHT-R and P300 in air. The rapid, initial desorption stage was removed in the weight loss graph to emphasize the difference between the two materials regarding thermal oxidation. The weight loss lines represent the mean of measurements of three specimens, except MHT-R at 1500 h, which is based on two measurements. The error bars are the standard deviation.

Figure 2a shows the weight loss behavior of the two polymer systems when aged at 288 °C. The weight that was lost during the first 4 h, due to the rapid initial desorption/drying, amounted to around 1% and was subtracted from the graphs. The overall observation was that the two formulations exhibited remarkably similar, almost identical, weight loss tendencies. This was attributed to, and confirmed, the large conformity in the formulations. Some minor differences worth noticing are that during the first days of ageing, the R300 showed a higher weight loss, while MHT-R displayed an induction period, where no weight loss was detected. The higher initial weight loss of R300 during the first 168 h of ageing is not fully understood but could be due to experimental uncertainties or residues, such as solvents, that were left within the material during manufacturing. Further studies are required to verify the reason for this behavior, for example, using mass spectrometry. Hence no significant difference in degradation tendency could be stated between the two formulations when comparing the results obtained from tests on bulk specimens, at the exposure times and temperature considered. Further studies with, e.g., thermal gravimetric analysis equipment should be performed on both materials to obtain data with a higher precision.

The weight loss was around 1% for both materials between the measurements at 4 and 500 h of ageing. The rate of weight loss increased between the 500 to 1000 h measurements and the weight loss was almost 3% during that interval, suggesting that a degradation mechanism different than thermal oxidation could have been initiated. One possible hypothesis is that thermal degradation through chain scission was accelerated during that period, perhaps as a result of the thermal oxidation. During the final interval between 1000 and 1500 h, the rate of weight loss decreased and was at the same level as it was during the first period up to 500 h.

**Figure 3:**
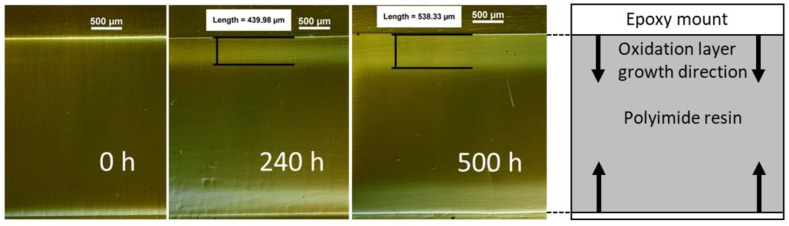
An example of oxidation layer measurements up to 500 h of ageing at 288 °C on R300 specimens. The images were obtained through reflected light DIC microscopy.

An oxidation layer was observed in both formulations after ageing. The layer thickness increased with ageing time (Figure 2b and Figure 3). Both material grades exhibited similar oxidation layer growth behavior. Initially, there was a rapid oxidation layer growth rate that eventually decreased until a steady state, constant, growth rate after 500 h was reached. The oxidation layer thickness is expected to be governed by the oxygen diffusion rate and the oxidation reaction rate and changes in both factors could be expected when modifying the chemistry of the resin systems. An increase in diffusion rate, resulting from the lower degree of crosslinking would mean that we could expect the oxidation layer to extend deeper into the material for the R300, which was not reflected in Figure 2. However, the effect from increased diffusion might be small enough to be either masked by the measurement uncertainty or counterbalanced by a corresponding decreased oxidation reaction rate in the formulation with fewer crosslinking sites.

It has been pointed out previously that in some polymer systems the oxidation layer could be difficult to distinguish with simple optical microscopy techniques [3]. In the current study, reflected light DIC microscopy was necessary to be able to render the oxidation layer visible. This technique is particularly useful for specimens with slight differences in height on the surface [15]. For the neat resin specimens used in this study, it was expected that the oxidation layer should have different properties than the bulk, resulting in different grinding/polishing rates for the two areas and, thus, differences in the morphology of the surface. The observed layer was diffuse, and a precise measurement of the extent of the oxidation layer was not feasible. Thus, the values of the oxidation layer thickness are to some extent approximate.

### 3.5. Nanoindentation

**Figure 4:**
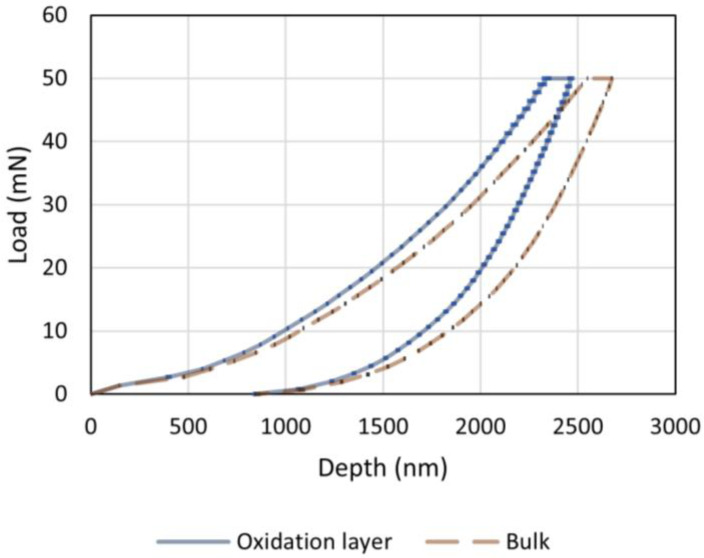
Nanoindentation measurements on an R300 polyimide after 500 h of ageing at 288 °C. The oxidation layer line is an average of 13 indentations and the bulk line is an average of five indentations. The horizontal bars in both lines represent the standard deviation. The largest bars in this figure represent 25 nm and 5 nm, respectively, for the oxidation layer and the bulk lines.

Figure 4 shows representative hysteresis curves from the nanoindentation measurements. The load-displacement data is presented as two curves, which were obtained by averaging the measurements for R300 after 500 h of ageing at 288 °C in the oxidation layer and the bulk, respectively. The curves consist of a loading segment up to 50 mN, which is followed by a dwell period, where the load is kept at a constant value of 50 mN and the maximum depth increases, and an unloading segment, where the load is brought down from 50 to 0 mN. A clear difference in the behavior was noticeable between measurements performed in the oxidation layer and measurements in the bulk, where no significant oxidation was expected.

**Figure 5:**
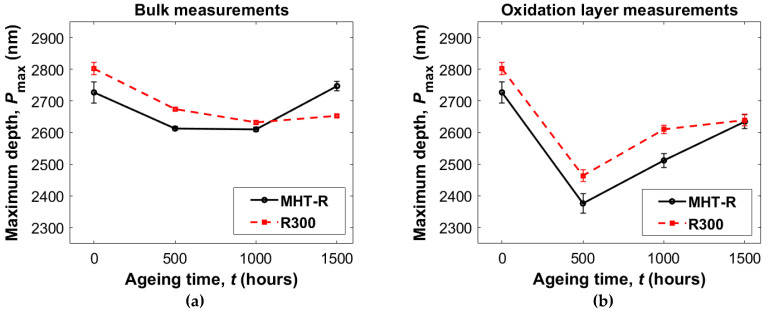
Maximum depth data from nanoindentation measurements in the bulk (**a**) and in the oxidation layer (**b**) of both formulations. The value for non-aged (0 h) material is the same in both graphs. The materials were aged at 288 °C in air. The bars represent standard deviation.

**Figure A2:**
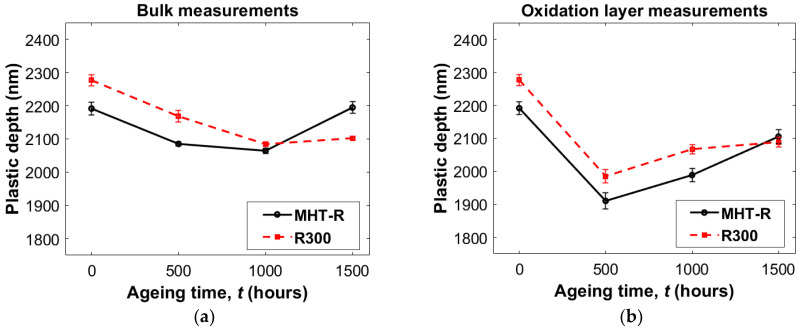
Plastic depth data from nanoindentation measurements in the bulk (**a**) and in the oxidation layer (**b**) of both formulations. The value for non-aged (0 h) material is the same in both graphs. The materials were aged at 288 °C in air. The bars represent standard deviation.

Figure 5 shows plots of the maximum depth in the bulk and in the oxidation layer at each ageing time. The remaining indentation depth after removing the load, or plastic depth, follows very closely the pattern of the maximum depth. This could be explained by the very short unloading time (2 s). The figures are attached in Appendix A, Figure A2. Comparing the non-aged materials, it could be noted that the indenter was able to penetrate deeper in the less crosslinked R300 PI than the MHT-R. This was also reflected in the initial average hardness (Table 3), where values of 0.373 GPa and 0.403 GPa were observed for R300 and MHT-R, respectively. After 500 h of ageing at 288 °C in air, the maximum penetration depth decreased, suggesting that the stiffness of the material has been increased, which could be a combined effect of additional crosslinking and oxidation. This was supported by the fact that the effect was more pronounced in the region close to the surface, where diffusion-governed oxidation was expected to occur. 

During the ageing period between 500 and 1500 h, the maximum depth started to follow a reversed pattern where the maximum depth was either constant or increased with increasing exposure. Increasing penetration depth for very long exposures (>500 h) is in this context taken as an indication that thermal degradation, and the associated chain scission reactions, is a primary degradation mechanism during long exposures. The reversed pattern was less pronounced in the bulk than the oxidation layer, suggesting that the oxidation layer could have reached the threshold for thermal degradation through chain scission faster than the bulk.

Young’s moduli values in the bulk and in the oxidation layer at different ageing times for both materials were summarized in Table 3. The average modulus before any ageing was similar for the two formulations, but slightly higher for MHT-R (4.64 GPa) than R300 (4.58 GPa). Because the difference was small, and due to the relatively large standard deviation in the MHT-R value (±0.130), it could not be concluded whether the slightly superior Young’s modulus of MHT-R was a result of the higher degree of crosslinking in that material. Overall, the moduli of the two formulations were not significantly different for all ageing times.

The modulus initially increased for both materials after ageing at 288 °C in air, peaking in the samples that were aged for 500 h (about 3 weeks), after which it decreased. The initial increase was observed both in the bulk and in the oxidation layer, where it was even more pronounced. The hardness and Young’s modulus obtained through nanoindentation in this study are similar to those reported for other thermosetting polyimides [7,8,15,16]. Contrary to what was previously observed in these studies, a decrease in the Young’s modulus in the interval between 500 and 1500 h of ageing was recorded in both MHT-R and R300. The mechanism which led to a decreased modulus at longer exposures is not completely understood at the moment, but one hypothesis is that it is a consequence of chain scission due to thermal degradation, as suggested earlier in the weight loss analysis. A complementary investigation, such as Fourier-transform infrared spectroscopy, could be performed on the oxidation layer to analyze the bond structure.

## 4. Conclusions

A new 6FDA-based polyimide formulation, R300, was introduced and compared to NEXIMID MHT-R. The goal was to reduce the cross-linking density, thus improving the fracture toughness. The study showed that there is a certain trade-off between the *T*g and the fracture toughness of the considered 6FDA-based polyimides. While a slight increase was noted in the average fracture toughness (0.88 MPa√m for R300 vs 0.78 MPa√m for MHT-R), the *T*g of the novel R300 was reduced by 27 °C. The formulations showed very similar thermal oxidation behaviors in the long-term ageing test, losing around 5% of their weight after 1500 h of exposure at 288 °C in air. After 500 h of ageing, both materials showed signs of accelerated weight loss, possibly due to thermal degradation through chain scission. Nanoindentation measurements showed that the hardness and the Young’s moduli of the two polyimides were similar before ageing. After 500 h of ageing, the materials experienced an increase in the properties in both the oxidation layer and the bulk. The increase was explained as a result of simultaneous oxidation and additional crosslinking in the polymers. When the materials were aged for up to 1500 h, a decrease in both properties was observed. It was hypothesized that the reduction could have been due to thermal degradation.

The measurements in this study were performed on bulk neat polymer specimens, except for the DSC tests, which were performed on pristine uncured resin powder. To fully understand the behavior and the consequences of the implemented changes, and whether the small difference in fracture toughness improves the matrix cracking, further studies on composite material would be required. Besides any potential difference in manufacturing-induced defects and cracks in composite plates, the mechanical properties should be further examined on composite level.

## Figures and Tables

**Table 1:** Fracture toughness data on the two polyimide systems.

	ID-number	K_IC_ [MPa√m]
	MHT-R (1)	0.85
	MHT-R (2)	0.80
	MHT-R (3)	0.70
**Average**	**MHT-R**	**0.78**
	R300 (1)	0.83
	R300 (2)	0.83
	R300 (3)	0.91
	R300 (4)	0.93
**Average**	**R300**	**0.88**

Note: MHT-R average was based on three measurements, and R300 on four.

**Table 2:** DSC and CTE data on the two polyimide systems.

ID-Number	Softening Point [°C]	Uncured *T*g [°C]	Heat of Reaction [J/g]	Cured *T*g [°C]	CTE [µm m^−1^ °C^−1^]
MHT-R (1)	121.5	116.9	282.5	437.4	56.8
MHT-R (2)	130.5	117.3	271.9	432.2	55.6
MHT-R (3)	119.8	119.8	308.3	427.5	55.6
**MHT-R (Average)**	**123.9**	**118.0**	**287.6**	**432.4**	**56.0**
R300 (1)	132.7	130.1	257.1	401.1	53.9
R300 (2)	132.0	130.4	267.8	405.4	54.0
R300 (3)	131.0	129.5	263.6	408.4	55.7
**R300 (Average)**	**131.9**	**130.3**	**262.7**	**405.0**	**54.3**

**Table 3:** Nanoindentation data on the two polyimide systems. Bulk values are based on 5 measurements in the middle (core) of the specimen, where the material should not have been affected by the ageing as much as the edge (shell) areas. Oxidation layer results are based on at least 10 measurements taken at approximately 50 µm from the edge.

		Bulk		Oxidation Layer	
Sample	Ageing Time [h]	Young’s Modulus [GPa]	Hardness [GPa]	Young’s Modulus [GPa]	Hardness [GPa]
MHT-R	0	4.64 (±0.130)	0.403 (±0.010)	N/A	N/A
MHT-R	500	4.94 (±0.020)	0.444 (±0.002)	6.10 (±0.183)	0.527 (±0.013)
MHT-R	1000	4.82 (±0.022)	0.453 (±0.003)	5.22 (±0.080)	0.487 (±0.010)
MHT-R	1500	4.49 (±0.086)	0.402 (±0.006)	4.88 (±0.061)	0.436 (±0.008)
R300	0	4.58 (±0.046)	0.373 (±0.006)	N/A	N/A
R300	500	4.96 (±0.177)	0.412 (±0.006)	5.72 (±0.104)	0.489 (±0.010)
R300	1000	4.76 (±0.017)	0.444 (±0.003)	4.84 (±0.040)	0.452 (±0.006)
R300	1500	4.69 (±0.018)	0.437 (±0.002)	4.72 (±0.072)	0.443 (±0.006)

Note: The values in parentheses are the standard deviation.

## Data Availability

Data sharing not applicable.
